# Efficient High-Rate Satellite Clock Estimation for PPP Ambiguity Resolution Using Carrier-Ranges

**DOI:** 10.3390/s141222300

**Published:** 2014-11-25

**Authors:** Hua Chen, Weiping Jiang, Maorong Ge, Jens Wickert, Harald Schuh

**Affiliations:** 1 School of Geodesy and Geomatics, Wuhan University, 129 Luoyu Road, Wuhan 430079, China; E-Mail: whuchenhua@gmail.com; 2 German Research Centre for Geosciences (GFZ), Telegrafenberg, Potsdam 14473, Germany; E-Mails: maor@gfz-potsdam.de (M.G.); wickert@gfz-potsdam.de (J.W.); schuh@gfz-potsdam.de (H.S.); 3 GNSS Research Center, Wuhan University, 129 Luoyu Road, Wuhan 430079, China

**Keywords:** precise point positioning service, high-rate clock correction, integer clock correction, carrier-range, integer ambiguity resolution

## Abstract

In order to catch up the short-term clock variation of GNSS satellites, clock corrections must be estimated and updated at a high-rate for Precise Point Positioning (PPP). This estimation is already very time-consuming for the GPS constellation only as a great number of ambiguities need to be simultaneously estimated. However, on the one hand better estimates are expected by including more stations, and on the other hand satellites from different GNSS systems must be processed integratively for a reliable multi-GNSS positioning service. To alleviate the heavy computational burden, epoch-differenced observations are always employed where ambiguities are eliminated. As the epoch-differenced method can only derive temporal clock changes which have to be aligned to the absolute clocks but always in a rather complicated way, in this paper, an efficient method for high-rate clock estimation is proposed using the concept of “carrier-range” realized by means of PPP with integer ambiguity resolution. Processing procedures for both post- and real-time processing are developed, respectively. The experimental validation shows that the computation time could be reduced to about one sixth of that of the existing methods for post-processing and less than 1 s for processing a single epoch of a network with about 200 stations in real-time mode after all ambiguities are fixed. This confirms that the proposed processing strategy will enable the high-rate clock estimation for future multi-GNSS networks in post-processing and possibly also in real-time mode.

## 1. Introduction

Precise point positioning (PPP), where precise orbit and clock corrections are essential [1], is widely applied in areas ranging from GNSS-based Precise Orbit Determination (POD) of Low Earth Orbit (LEO) satellites to ground surveying. Due to the high altitude of GNSS satellites, their orbits can be precisely determined and predicted as well. Therefore, orbits do not have to be updated very fast, and even predicted orbits can also fulfill the PPP requirement for a few hours. However, satellite clock corrections must be estimated or updated very frequently due to their short-term variations which are confirmed by the significant difference between estimated high-rate corrections and values interpolated from low-rate estimates [2]. Montenbruck *et al.* [3] proved that the linearly interpolated clock corrections from 5-min clock products are not sufficient to achieve high LEO orbits accuracy of a few cm. Therefore, 30 s or even higher rate satellite clock corrections are necessary to PPP for precise applications, in particular for kinematic applications such as orbit and trajectory determination of LEO and other moving platforms.

Usually, phase ambiguities must be estimated simultaneously with clock corrections using phase and range observations from a global GNSS ground network. The large number of ambiguities makes the estimation of high-rate clock corrections quite time-consuming, what is particularly significant for real-time applications. This situation will become even worse as more satellites and stations are included in the processing. On the one hand, processing of denser networks is suggested for better clock corrections. On the other hand, newly emerging GNSS systems, such as the Chinese Beidou system (BDS) and the European GALILEO system, are expected to be involved in current GPS and GLONASS processing to provide multi-GNSS services for better accuracy and reliability.

In order to improve the computational efficiency, several approaches have been developed for estimating high-rate clocks by using epoch-differenced observations where ambiguities are removed. For post-processing, Bock *et al.* [2] developed an efficient algorithm to estimate clocks of 30 s and/or 5 s based on the low-rate clock corrections (e.g., 300 s) from a regular orbit determination procedure. In the algorithm, clock differences between adjacent epochs are estimated using epoch-differenced phase observations and then the estimates are combined with the low-rate clocks as control points to obtain the final high-rate clock corrections. For real-time processing, Zhang *et al.* [4] and Ge *et al.* [5] suggested a similar processing strategy with two processes running in parallel. One is to estimate clock corrections directly, but with a slow update rate of about 300 s, while the other one is to estimate epoch-differenced clock corrections with a high update rate. The epoch-differenced clocks are converted to high-rate clocks with the data provided by the first process. Although clock corrections from the epoch-differenced solutions are of high quality and in principle could satisfy the demands of PPP applications, the absolute clocks are maintained in a complicated way, especially in the real-time mode. In addition, this solution is theoretically different from the undifferenced solutions, because correlations between epochs are neglected.

Instead of the epoch-differenced method, the concept of “carrier-range” proposed by Blewitt *et al.* [6] provides a new perspective to relieve the computational burden, where all the ambiguities are resolved in advance [7]. Based on the carrier-range concept, Chen *et al.* [8] use PPP with ambiguity resolution to produce carrier-ranges on station base for efficiently processing huge networks. In this paper, the method is modified for estimating high-rate clock corrections and two processing procedures are developed for both post-processing mode and real-time mode, respectively. In the post-processing mode, the fixed integer ambiguities based on the low-rate products are properly introduced to convert high-rate phases to carrier-ranges. In contrast, the fixed integer ambiguities of the previous epoch are used for generating carrier-ranges at the current epoch in real-time mode. In this way, the clock corrections can be estimated very efficiently because no or only a few ambiguities are included in the estimation.

In the following sections, the current methods using undifferenced and epoch-differenced observations are firstly introduced; and then the new processing procedures based on carrier-range for post-processing mode and real-time mode are presented, respectively. Finally the computation efficiency and the quality of clock corrections from the new processing procedure are accessed.

## 2. Undifferenced Estimation

As station coordinates, satellite orbits and Earth Rotation Parameters (ERP) can be well determined in advance, and they are usually fixed in the clock estimation. For example, for estimating high-rate clocks in post-processing mode, the above-mentioned parameters and zenith total delay (ZTD) as well, are already precisely estimated in the precise orbit determination with a lower sampling rate. Furthermore, the ZTD parameters could also be fixed in the post-processing mode, whereas in the real-time mode, they must be estimated with clock corrections together. Therefore, the observation equations of ionosphere-free combination can be expressed as:

(1) $$\begin{array}{c} v_{Lc}=\delta \;t_{r}-\delta t_{s}+m\delta T+\lambda_{1}b_{c}+l_{Lc}\\ v_{Pc}=\delta \;t_{r}-\delta t_{s}+m\delta T+l_{Pc} \end{array}$$

where, δ*t_r_* and δ*t_s_* are the receiver and satellite clock, respectively; δ*T* and *m* are ZTD and the corresponding mapping function; *b_c_* represents the phase ambiguities; *l_Lc_* and *l_Pc_* are pre-fit residuals of the phase and range observations, while *v_Lc_* and *v_Pc_* represent the related post-fit residuals, respectively. In addition, the phase center correction and the phase windup effect must be corrected in modeling and the satellite-dependent differential code biases (DCB) [9] should also be applied, especially if different types of range observations are employed.

The estimation using Equation (1) is nowadays widely applied to network solutions, such as precise orbit determination, and it is rather time-consuming due to the great number of ambiguities. The number of ambiguities increases dramatically along with the number of stations and satellites, so that it is very difficult to carry out for large networks and for multi-GNSS constellations. In order to improve the computational efficiency, epoch-differenced methods are proposed where ambiguities are eliminated.

## 3. Epoch-Differenced Estimation

Based on the undifferenced observation equations Equation (1), the epoch-differenced observation equations can be written as:

(2) $$\begin{array}{c} v_{\Delta Lc}\left(i\right)=\Delta \delta t_{r}\left(i\right)-\Delta \delta t_{s}\left(i\right)+\Delta m\left(i\right)\Delta \delta T\left(i\right)+\Delta l_{Lc}\left(i\right)\\ v_{\Delta Pc}\left(i\right)=\Delta \delta t_{r}\left(i\right)-\Delta \delta t_{s}\left(i\right)+\Delta m\left(i\right)\Delta \delta T\left(i\right)+\Delta l_{Pc}\left(i\right) \end{array}$$

where, Δ is the differential operator between two adjacent epochs, for example, the differenced clock correction is Δδ*t_s_* (*i*) = δ*t_s_* (*i*) − δ*t_s_* (*i*−1).

As ambiguities are removed from observation equations, the computational efficiency could be improved significantly, and the epoch-differenced clocks can also be estimated precisely. However, the clock correction at the initial epoch is required in order to obtain the clock corrections by the accumulation of the successive epoch-differenced clock corrections. The maintenance of the absolute clock offsets thus becomes a critical issue in the epoch-differenced methods. For the post-processing step, Bock *et al.* [2] proposed the use of the estimated low-rate clocks as control points to align the accumulated clock changes to absolute clocks. For real-time processing, Zhang *et al.* [4] and Ge *et al.* [5] suggested a similar processing schema with two parallel processes—one is to estimate the epoch-differenced clocks and the other is to estimate the clock offsets. In all the above methods, if cycle slips happen, no epoch-differenced observations could be formed with respect to the last epoch. In the case that most of the stations lose tracking of one satellite, its absolute clock offset cannot be propagated forward. Furthermore, using epoch-differenced observations is not theoretically equivalent to that using undifferenced ones, as the statistical correlations between differenced observations is usually neglected.

## 4. Carrier-Range Methods

Similar to the method for huge network solutions [8], the ambiguities could be resolved station by station in advance. For completeness, we briefly introduce the carrier-range method here. In Equation (1), the ambiguities of the ionosphere-free combination are always expressed as wide-lane *b_w_* and narrow-lane *b_n_* for ambiguity resolution:

(3) $$b_{c}=\frac{f_{1}}{f_{1}+f_{2}}b_{n}+\frac{f_{1}f_{2}}{f_{1}^{2}-f_{2}^{2}}b_{w}$$

where *b_c_* is the ambiguity of ionosphere-free combination; *f*_1_ and *f*_2_ are signal frequencies; *b_w_* and *b_n_* are wide-lane and narrow-lane ambiguities, respectively. Although *b_w_* and *b_n_* are not natural integer values due to the existence of the uncalibrated phase delays (UPD), the fractional part of UPDs can be estimated from a reference network and applied to all the stations for integer ambiguity resolution in PPP mode [10].

The wide-lane *b_w_* could be calculated from Melbourne-Wübbena combination [11,12], then, similar to [13,14], the fractional part of *b_w_* could be expressed as:

(4) $$Fb_{w}=\delta b_{wr}+\delta b_{w}^{s}$$

where *Fb_w_* is the fractional part of wide-lane and δ*b_wr_*, $\delta b_{w}^{s}$ represent receiver-dependent and satellite-dependent wide-lane UPDs. After fixing a receiver-dependent UPD or a satellite-dependent UPD, wide-lane UPDs could be derived from Equation (4).

With the help of wide-lane UPDs, wide-lane ambiguities could be fixed. Afterwards, *b_w_* can be replaced by its integer number, then the related wide-lane UPDs could be merged into the associated narrow-lane ambiguity. Therefore Equation (3) can be written as:

(5) $$b_{c}=\frac{f_{1}}{f_{1}+f_{2}}b_{n}^{\prime }+\frac{f_{1}f_{2}}{f_{1}^{2}-f_{2}^{2}}N_{w}$$

(6) $$b_{n}^{\prime }=N_{n}+\delta b_{nr}+\delta b_{n}^{s}$$

where $b_{n}^{\prime }$ is the new narrow-lane ambiguities after absorbing the related wide-lane UPDs; *N_w_* is the fixed integer value of the wide-lane ambiguity; *N_n_*, δ*b_nr_* and $\delta b_{n}^{s}$ are the integer narrow-lane ambiguity and the related UPD for the receiver and satellite, respectively.

From Equation (5), the new narrow-lane could be derived from a known ionosphere-free ambiguity and a relative fixed wide-lane ambiguity. Then similar to the calculation of wide-lane UPDs, the fractional part of the new narrow-lane $b_{n}^{\prime }$ could be expressed as:

(7) $$Fb_{n}^{\prime }=\delta b_{nr}+\delta b_{n}^{s}$$

where $Fb_{n}^{\prime }$ is the fractional part of narrow-lane and δ*b_nr_*, $\delta b_{n}^{s}$ represent receiver-dependent and satellite-dependent narrow-lane UPDs.

According to the above-mentioned approach, the wide-lane UPDs and narrow-lane UPDs could be derived accurately and applied to any other station to recover the integer nature of ambiguities for fixing. After both the wide-lane and narrow-lane ambiguities are fixed, taking Equations (5) and (6) into consideration, the observation equation Equation (1) could be rewritten as:

(8) $$\begin{array}{l} v_{Lc}-\lambda_{1}\left(\frac{f_{1}}{f_{1}+f_{2}}N_{n}+\frac{f_{1}f_{2}}{f_{1}^{2}-f_{2}^{2}}N_{w}\right)=\delta t_{r}-\delta t_{s}+\lambda_{1}\frac{f_{1}}{f_{1}+f_{2}}\left(\delta b_{nr}+\delta b_{n}^{s}\right)+m\delta T+l_{Lc}\\ v_{Pc}=\delta t_{r}-\delta t_{s}+m\delta T+l_{Pc} \end{array}$$

where the integer ambiguities are introduced into the equations as known values. This also means that the observations correspond to an ambiguity-fixed solution and will certainly result into better clock estimates if there is no wrong fixing. Of course, in practice, it is hard to fix all the ambiguities correctly, especially for PPP where UPDs bring additional errors. In this paper, we simulated the real-time processing and with a focus on its computational efficiency, in real data processing, we suggest to only fix those ambiguities with high confidence. It should also be pointed out that the narrow-lane UPDs at the receiver and satellite can either be corrected or estimated. In principle, the narrow-lane UPD parameters cannot be ignored, as they could be absorbed by the clock parameters of the carrier-range observation equation and that will lead to inconsistent definition of clock parameters in the carrier-range and code-range observations. If only carrier-range observations are used for clock correction determination, then UPDs cannot be separated from clock corrections, the Equation (8) can be written as:

(9) $$\begin{array}{l} v_{Lc}-\lambda_{1}\left(\frac{f_{1}}{f_{1}+f_{2}}N_{n}+\frac{f_{1}f_{2}}{f_{1}^{2}-f_{2}^{2}}n_{w}\right)=\delta t_{ir}-\delta ti_{s}+m\delta T+l_{Lc}\\ \delta t_{ir}=\delta t_{r}+\lambda_{1}\frac{f_{1}}{f_{1}+f_{2}}\delta b_{nr}\\ \delta t_{is}=\delta t_{s}+\lambda_{1}\frac{f_{1}}{f_{1}+f_{2}}\delta b_{n}^{s} \end{array}$$

where δ*t_ir_* and δ*t_is_* are the receiver and satellites clocks including the corresponding UPDs, respectively. With these clock corrections, the estimated ambiguities will have integer characteristics, thus they are also referred to as integer clocks [15,16]. In principle, the method could be directly applied for data processing of three frequencies if two ionosphere-free observations are performed. However, recent studies showed the possible inconsistency of GPS L5 with L1 and L2 [17]. Therefore, there are still issues that should be investigated, especially the DCBs, UPDs and their consistency. This is also the reason we only include two frequencies in this paper.

**Figure 1.**
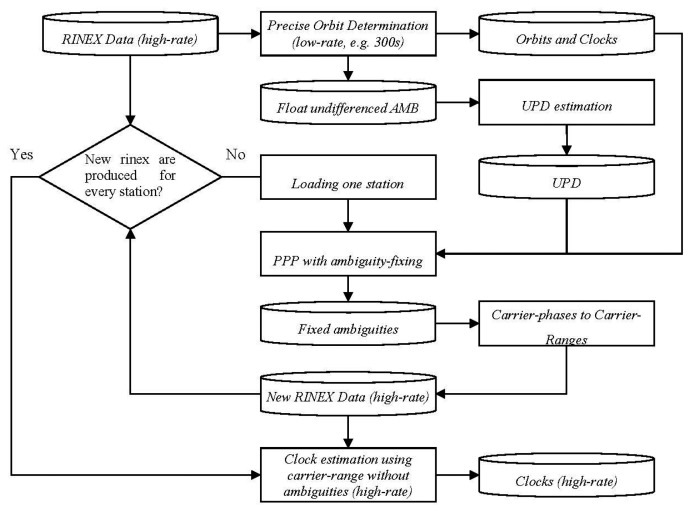
Overview on the estimation of clock corrections in post-processing mode using carrier-ranges.

**Figure 2.**
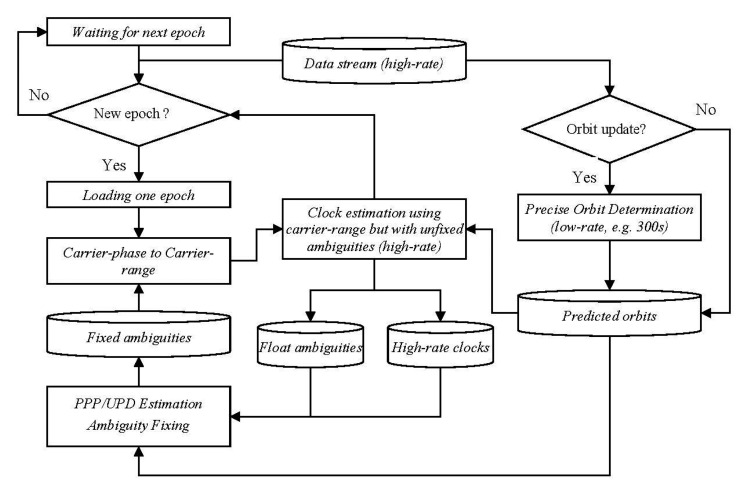
Overview on the estimation of clock corrections in real-time mode using carrier-range.

Since there are no ambiguities to be estimated for the carrier-range, the efficiency could be improved greatly. Taking all the above into consideration, the processing procedures using the carrier-range concept can be illustrated in Figures 1 and 2 for post-processing and real-time processing, respectively.

As illustrated in Figure 1, the estimation of clock corrections in post-processing mode can be carried out with the following steps:

(1) Precise orbit determination with a low sampling rate (e.g., 300 s) is carried out to provide orbits and clocks similar to the IGS final products using a global network with homogeneously distributed stations.
(2) UPDs are derived from the float ambiguities in precise orbit determination according to Equations (4) and (7) and the magnitude of UPDs is kept below one cycle for both wide-lane and narrow-lane.
(3) The RINEX files are loaded one by one, and PPP with ambiguity fixing is carried out station by station with the help of the precise orbits, clocks (low-rate) and UPDs. The fixed ambiguities are stored in a station-related file.
(4) With the fixed ambiguities, a new RINEX file is created by converting the carrier-phases of the original RINEX file into carrier-ranges. The fixed ambiguities are also applied for those epochs which are not used in the low-rate processing, if no cycle slip occurs. Therefore the sampling interval of the new RINEX file is kept the same as the original one.
(5) Repeat steps (3) and (4) until there is a related new RINEX file for each station.
(6) Based on the new RINEX files, clock corrections or integer clock corrections can be achieved using the efficient way expressed by Equations (5) and (6), respectively.

Similarly, as shown in Figure 2, the whole procedure of the real-time processing can be described as follows:

(1) Similar to the post-processing mode, precise orbit determination with a processing interval of 300 s is carried out in batch mode with a fixed update rate, for example every few hours, and the predicted orbits are generated for clock estimation.
(2) The clock estimation is carried out according to Equation (1) with the predicted orbits. At the beginning, all ambiguities must be estimated. During this period, sampling rate and stations involved could be properly reduced in order to save computation time.
(3) The estimated undifferenced ambiguities can be utilized for UPD estimation for undifferenced ambiguity resolution. Here, we can also perform PPP for each of the reference stations and to estimate UPDs with the PPP estimated ambiguities
(4) The fixed ambiguities are added to the phase observations of the consequent epochs to obtain carrier-ranges if no cycle slips were detected. These carrier-ranges will be used in step 2 for high-rate clock estimation for saving computational time.
(5) Steps (2)–(4) is carried out for each epoch. As more and more ambiguities could be fixed along with the epoch-increasing progress, the processing is accelerated epoch by epoch.

## 5. Experimental Validation

As the critical task of this study is to tackle the heavy computation burden for the clock estimation, the major aim of the experimental validation is the assessment of the computational efficiency of the new approach.

### 5.1. Software and Data

**Figure 3.**
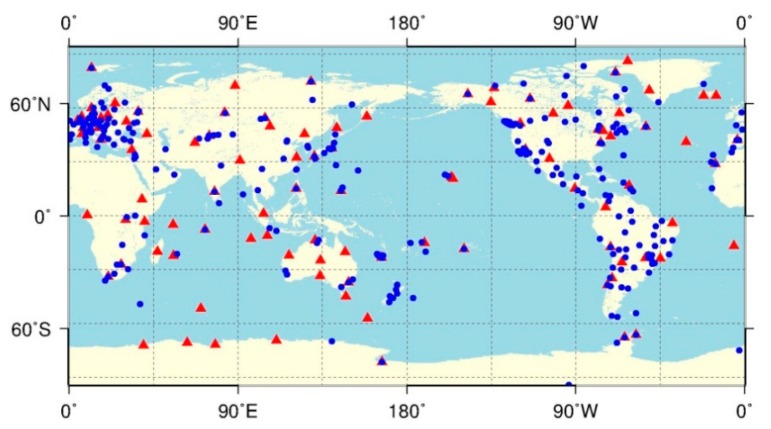
Station distribution of the experimental network. There are about 450 stations in total. Among them, about 100 stations indicated with red triangles, are used for orbit determination and high-rate clock estimation, while the blue dots indicate the other about 350 stations for assessing product quality.

In order to validate the new processing method based on the carrier-range concept, the PANDA software developed at Wuhan University [18,19] was adapted for processing carrier-ranges. Two processing procedures for post-processing and real-time processing were developed with the improved version of PANDA as processing core. GPS data with a sampling rate of 30 s from about 450 global distributed permanent stations, as shown in Figure 3, over the time from DOY 203 to 238, 2012, were employed. Different networks would be defined for various processing scenarios.

### 5.2. Processing Scenarios

In order to assess the efficiency of the carrier-range strategy, five networks with station number of 50, 100, 150, 200, and 250 were defined. Data on the day 203, 2012 of all the networks were processed using the undifferenced method and the carrier-range method in the post-processing and real-time mode, respectively. The sampling rate was 30 s in order to obtain the high-rate clock corrections. The computational time of one least-squares adjustment for each network was recorded and compared for assessing the computational efficiency of each method.

For post-processing, the traditional undifferenced method was rather straightforward following the approach of Ge *et al.* [20], where ambiguities were estimated as unknowns and only active parameters were kept in the normal equation to save computation time. The carrier-range method was carried out following the procedure illustrated in Figure 1. In the first step, the orbits and clocks of 300 s interval were estimated using the network with about 100 reference stations (indicated by red triangles in Figure 3) for all the above mentioned networks.

For the real-time mode, simulated real-time processing was undertaken and we even assumed that all ambiguities were resolved in advance to test the computation time. According to [21], PPP ambiguity-resolution could be carried out stably with a high success rate, so this simulation is reasonable to test the possible efficiency this strategy could achieve in real time. In other words, the raw data were read from RINEX files and then the fixed ambiguities were employed to obtain carrier-ranges. Afterwards, the clocks were estimated epoch by epoch with ZTD parameters, simultaneously. The average computational times for one single epoch of both methods were compared.

Additionally, the orbits and high-rate clocks were also briefly assessed but only for the post-processing products. Among the 450 IGS stations shown in Figure 3, about 100 core stations indicated with red triangles were used to generate the satellite clocks, while the remaining indicated by blue dots were employed as PPP user stations. For each day from 203 to 238, 2012, the 30 s clocks were estimated following the procedure of Figure 1. In the final step only carrier-range measurements were adopted, so that with this kind of products, ambiguity fixing could be performed directly without satellite UPDs. Then PPP was carried out with the orbits and high-rate clocks for the 350 user stations and with ambiguity fixing. The results both before and after ambiguity fixing were compared to the IGS weekly solutions.

### 5.3. Validation of Computational Efficiency

**Figure 4.**
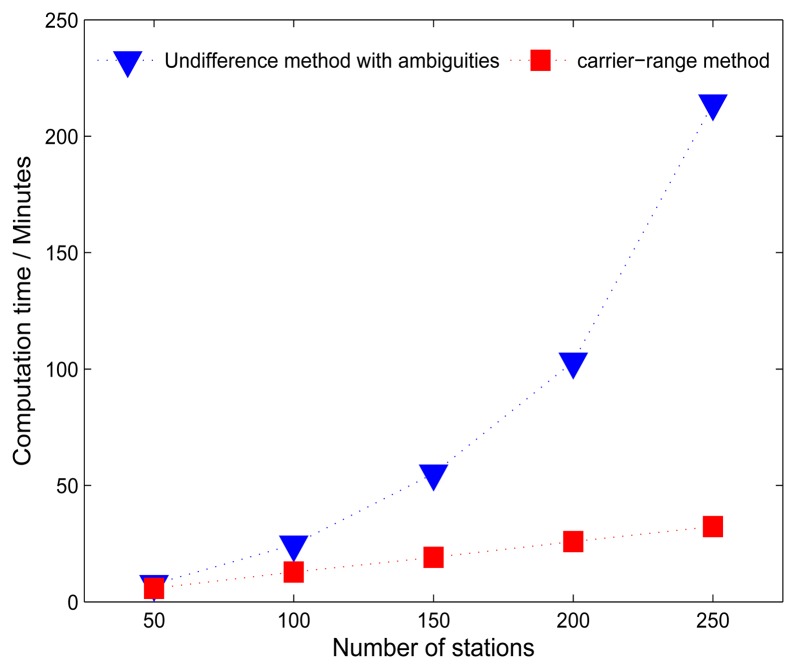
Comparison of computation times of the new method (red squares) and traditional method (blue triangles) for networks with different number of stations.

All the test computations were carried out on a computer equipped with two processors (2.13 GHZ) and 4 GB RAM under openSUSE linux operation system. For post-processing, as the orbits and clocks of a low-rate processing are already available, PPP is usually employed to speed up the data cleaning. This means, in both the traditional method and the carrier-range method, PPP is iteratively carried out for all the involved stations. The additional steps for the carrier-range method are the UPD estimation, PPP ambiguity-fixing and generation of RINEX files with carrier-ranges. However, UPD estimation is quite efficient, it takes only about 50 s for the daily data from a network with about 100 stations and the UPD estimation only needs to be estimated once a day. Other steps can be carried out station by station in parallel and it takes only about 5 s to finish all these steps for one station. Therefore, only the computation time for the final step is considered here. As shown in Figure 4, the carrier-range method has a linearly increasing processing time proportional to the increasing number of stations. It only takes 32 min for processing a network with about 250 stations, which saves about 85% computation time compared to the traditional ones. The results also suggest that more stations and more satellites could be included in the carrier-range method.

**Figure 5.**
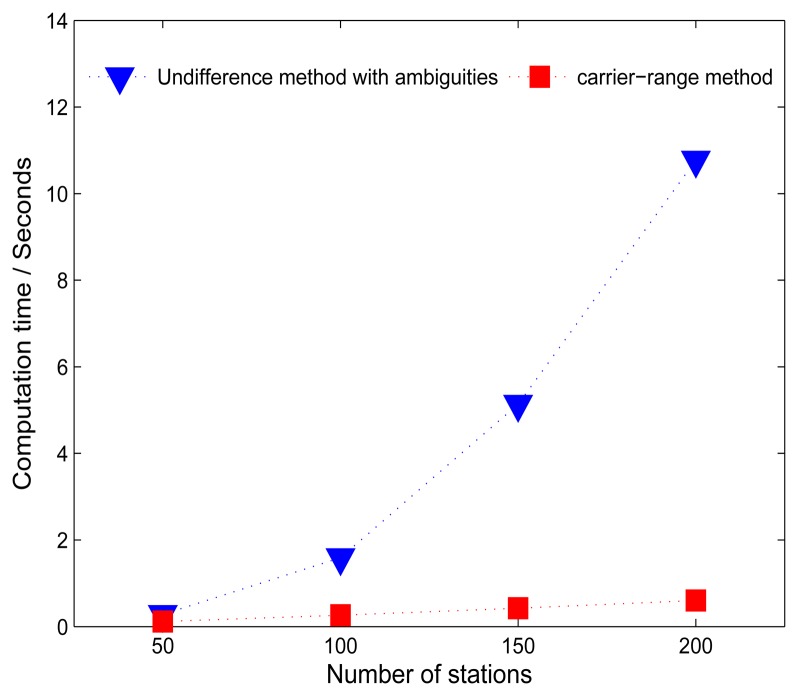
Comparison of average computation time per epoch of the new method (red squares) and traditional method (blue triangles) for networks with different number of stations.

For real-time processing, average computation time per epoch of both simulated experiments (carrier-range method) described above and the undifferenced method for networks with different number of stations, are shown in Figure 5. It can be seen that it takes only 0.12, 0.26, 0.42 and 0.60 s per epoch for networks comprising 50, 100, 150 and 200 stations, respectively, whereas, 10.7 s is needed for the traditional method for the network comprising 200 stations. The computation time of the carrier-range method has a linear increase along with the increase of the number of stations. This also demonstrates the potential of including more stations and satellites for clock estimation in real-time with the new strategy.

### 5.4. Clock Quality

Since the real-time experiments are simulated only for testing the possible efficiency this method could achieve, and its fixed ambiguities are also from post-processing, in this paper, only clocks from post-processing are tested for quality checking. Statistically, about 94% narrow-lane ambiguities over the experimental period for all stations could be fixed, and we found that most of the unfixed ambiguities are related to a low elevation angle, which shows a great consistency between clock corrections and ambiguities.

The difference of the station coordinates between PPP results and IGS weekly solution provides a more realistic indicator of the quality of the integer clocks. As shown in Table 1, after having applied a seven-parameter transformation, the mean RMS of ambiguity-float solutions in east, north and up directions are 3.8, 3.0 and 6.0 mm, respectively, while those for ambiguity-fixed solutions are 2.6, 2.9 and 5.8 mm, respectively. The ambiguity resolution mainly contributes to the east components, which coincides with the foundings by Geng *et al.* [22].

## 6. Conclusions

Two processing procedures for estimating high-rate clock corrections for post-processing and real-time processing, respectively, are developed using the carrier-range observations generated based on PPP with ambiguity resolution. In post-processing, PPP is firstly carried out at every station at low rate processing interval, and the ambiguities are resolved after applying UPD corrections. Then all the carrier-phases are converted to carrier-ranges with a high-rate sampling, and finally, with the carrier-ranges the clocks determination can be done in an efficient way because no ambiguities or only few unfixed ambiguities need to be estimated. In real-time mode, the whole procedure starts with the undifferenced model. Then, the fixed ambiguities from last epoch are used to convert the carrier-phases to carrier-ranges at current epoch if no cycle slips were detected. The processing is accelerated step by step by fixing more and more ambiguities.

A preliminary validation experiment shows that it takes only about 32 min for a network with about 250 stations for estimating clocks of 30 s sampling rate, while it takes about 214 min using the traditional method for daily data.

With the estimated integer clocks, about 94% ambiguities could be fixed in the PPP processing. The mean RMS of station coordinates of the PPP fixed solutions in east, north and up directions with respect to IGS weekly solution are 2.6, 2.9 and 5.8 mm, respectively. For real-time processing, the simulated experiment shows that it takes only 0.12, 0.26, 0.42 and 0.6 s for a single epoch for networks comprising of 50, 100, 150 and 200 stations.

The experimental validation confirms that the new processing strategy will enable the high-rate clock estimation for future multi-GNSS networks in post-processing and possibly also in real-time mode.

## Figures and Tables

**Table 1.** Mean RMS of station coordinates with respect to IGS weekly solution.

**Type (the Processing Interval is 30 s)**	**Mean RMS with Respect to IGS Weekly Solutions (mm)**		

	**East**	**North**	**Up**
Ambiguity-float solutions	3.8	3.0	6.0
Ambiguity-fixed solutions	2.6	2.9	5.8
